# Artificial Intelligence Meets Dementia Care: Co-Production for Accessibility and Inclusivity in the LUMEN Project

**DOI:** 10.1192/bjo.2026.11236

**Published:** 2026-06-30

**Authors:** Mohamed Elsharif, Indiana Varley, Niema Moazzami, John-Paul Taylor, Judith Rose Harrison

**Affiliations:** 1 Cumbria, Northumberland, Tyne and Wear NHS Foundation Trust, Newcastle upon Tyne, United Kingdom; 2 Newcastle University, Newcastle upon Tyne, United Kingdom; 3 Cumbria,Northumberland, Tyne and Wear NHS Foundation Trust, Newcastle uponTyne, United Kingdom; 4 Campus for Ageing and Vitality Newcastle University, Newcastle upon Tyne, United Kingdom

## Abstract

**Aims:**

Artificial intelligence (AI) possesses the capacity to fundamentally alter the landscape of dementia care. Nevertheless, for such instruments to achieve efficacy, they must be meticulously crafted to accommodate the heterogeneous requirements of patients, caregivers, and healthcare practitioners. The LUMEN project (Large Language Model for Understanding and Monitoring Elderly Neurocognition) is in the process of developing an AI-assisted instrument for dementia evaluation, which employs a Large Language Model to derive structured collateral histories from relatives or caregivers of patients. The co-production with stakeholders is paramount to affirming that LUMEN is not merely clinically efficacious but also user-centred and culturally pertinent across varying demographic groups.

**Methods:**

A succession of two co-production workshops has been executed with caregivers, and patient groups representatives. Participants have been recruited from a spectrum of cultural, linguistic, and digital backgrounds, with deliberate partnerships established with community organizations, particularly those representing underserved populations. These workshops concentrated on assessing LUMEN’s interface, linguistic clarity, and cultural relevance. Participants interacted with the LUMEN prototype, offering feedback regarding language, interface functionality, and overall user experience. Employing a ‘Think Aloud’ methodology, participants vocalized their immediate reactions during their interaction with the tool, enabling facilitators to gather valuable data concerning usability and engagement. Feedback was audio recorded, transcribed, and systematically analysed through thematic analysis, thus identifying critical themes and patterns that illuminate challenges pertaining to language, interface design, and cultural sensitivity.

**Results:**

Ten themes and 44 sub-themes were identified, most relating to language accessibility, question design, cultural and social appropriateness, role assumptions, and system usability. Over one-third of sub-themes were rated high priority and almost 90% were deemed actionable, indicating substantial scope for redesign. Participants highlighted medical jargon, compound and ambiguous questions, culturally biased assumptions (for example, gender roles and technology use), unclear intended user (self vs carer), and rigid response formats as key barriers to acceptability. Strengths included the potential to complete LUMEN at home, inclusion of carer wellbeing, and capacity to capture nuanced information when free-text fields worked well.

**Conclusion:**

Co-production with people living with dementia, carers, and professionals revealed that LUMEN’s acceptability depends on simplifying language, clarifying question framing around change from baseline, improving usability, and culturally adapting content. The high proportion of actionable findings demonstrates the practical value of think-aloud co-production for optimising AI-enabled dementia assessments and provides a roadmap for iterative redesign towards more equitable, user-centred tools.

